# Epigenomic plasticity of Arabidopsis msh1 mutants under prolonged cold stress

**DOI:** 10.1002/pld3.79

**Published:** 2018-08-29

**Authors:** Sunil Kumar Kenchanmane Raju, Mon‐Ray Shao, Yashitola Wamboldt, Sally Mackenzie

**Affiliations:** ^1^ Department of Agronomy and Horticulture University of Nebraska‐Lincoln Lincoln Nebraska; ^2^ Present address: Departments of Biology and Plant Science Pennsylvania State University University Park Pennsylvania

**Keywords:** abiotic stress, DNA methylation, phenotypic plasticity

## Abstract

Dynamic transcriptional and epigenetic changes enable rapid adaptive benefit to environmental fluctuations. However, the underlying mechanisms and the extent to which this occurs are not well known. *MutS Homolog 1* (*MSH1*) mutants cause heritable developmental phenotypes accompanied by modulation of defense, phytohormone, stress‐response, and circadian rhythm genes, as well as heritable changes in DNA methylation patterns. Consistent with gene expression changes, *msh1* mutants display enhanced tolerance for abiotic stress including drought and salt stress, while showing increased susceptibility to freezing temperatures. Despite changes in defense and biotic stress‐response genes, msh1 mutants showed increasing susceptibility to the bacterial pathogen *Pseudomonas syringae*. Our results suggest that chronic cold and low light stress (10°C, 150 μmol m^−2^ s^−1^) influences non‐CG methylation to a greater degree in *msh1* mutants compared to wild‐type Col‐0. Furthermore, CHG changes are more closely pericentromeric, whereas CHH changes are generally more dispersed. This increased variation in non‐CG methylation pattern does not significantly affect the *msh1*‐derived enhanced growth behavior after mutants are crossed with isogenic wild type, reiterating the importance of CG methylation changes in *msh1*‐derived enhanced vigor. These results indicate that *msh1*methylome is hyper‐responsive to environmental stress in a manner distinct from the wild‐type response, but CG methylation changes are potentially responsible for growth vigor changes in the crossed progeny.

## 1 INTRODUCTION

Plants have developed mechanisms to overcome constantly changing environments. Species that are more adaptable to changing environments through phenotypic plasticity and selection of adaptable traits survive. These changes occur at different levels, from morphological and physiological changes to modulations in gene expression and chromatin behavior, allowing plants to cope with the challenges of nature. While a major source of this adaptive response can be attributed to genetic variation (Franks & Hoffmann, 2012), recent studies are pointing toward the potential role of chromatin modifications and epigenetics in plant responses to environmental changes (Bilichak & Kovalchuk, 2016). Environment‐induced epigenetic modifications are generally transient, and the consistency of the environmental cue perceived by plants plays a role in inducing epigenetic changes and their inheritance (Uller, English, & Pen, 2015).

Cytosine DNA methylation is a heritable epigenetic modification involving the addition of a methyl (‐CH3) group to the fifth carbon of the pyrimidine ring of cytosine nucleotides. This addition is catalyzed by DNA methyltransferases, commonly found in most eukaryotes (Cheng, 1995). In plants, DNA methylation can occur in three sequence contexts: the symmetric CG and CHG contexts, and the asymmetric CHH context, where H represents A, C, or T nucleotides (Law & Jacobsen, 2010). Methylation in these different contexts displays distinct genomic patterning within genes, repeat regions, and transposable elements. While CG methylation is largely concentrated within genes and transposable elements, CHG and CHH methylation contexts are usually associated with repeat regions and transposable elements (Cokus et al., 2008).

One role of DNA methylation is to silence transposable elements, which can become activated during stress conditions (Slotkin & Martienssen, 2007). In some cases, changes in DNA methylation have also been associated with stress‐induced gene regulation, such as during phosphate starvation or *Pseudomonas syringae* infection (Dowen et al., 2012; Yong‐Villalobos et al., 2015) and may provide the mechanistic basis for memory (Dowen et al., 2012; Kinoshita & Seki, 2014). Despite major progress in dissecting the genetic pathways responsible for establishment and maintenance of context‐specific DNA methylation patterns (Stroud, Greenberg, Feng, Bernatavichute, & Jacobsen, 2013), functions of DNA methylation, particularly genic CG methylation, have remained mysterious (Zilberman, 2017).

*MutS Homolog 1* (*MSH1*) is a plant‐specific, nuclear‐encoded gene that targets its protein to both plastids and mitochondria. Arabidopsis *msh1* mutants display a range of altered phenotypes that include variegation, dwarfing, delayed maturity transition, delayed flowering, and partial male sterility (Xu et al., 2011). The T‐DNA *msh1* mutants display higher tolerance to heat, high light, and drought stress (Shedge, Davila, Arrieta‐Montiel, Mohammed, & Mackenzie, 2010; Virdi et al., 2016; Xu et al., 2011), particularly in individuals showing stronger developmental phenotypes. *MSH1* phenotypes are conserved between monocots and eudicots. This conservation is evidenced in the RNAi suppression phenotypes, and the consistent observation that subsequent MSH1‐RNAi transgene segregation gives rise to trans‐generational *msh1* memory in sorghum, pearl millet, tomato, tobacco, and soybean (de la Rosa Santamaria et al., 2014; Kenchanmane Raju et al., 2018; Xu et al., 2011, 2012; Yang et al., 2015). These memory lines show attenuated *msh1* phenotype, even though *MSH1* transcripts are back to wild‐type levels (Raju et al., 2018).

Disruption of *MSH1* causes genome‐wide methylome repatterning in both CG and non‐CG context (Virdi et al., 2015), along with large‐scale changes in gene expression related to abiotic and biotic stress response, phytohormone pathways, circadian rhythm, defense response, and signaling (Shao, Kumar Kenchanmane Raju, Laurie, Sanchez, & Mackenzie, 2017). Arabidopsis *msh1* memory lines show a subset (ca 10%) of the gene expression changes of the T‐DNA insertion mutant, with enrichment in circadian rhythm, ABA signaling, and light‐response pathways, and with methylome repatterning predominantly in CG context (Sanchez et al., 2018).

In this study, we investigated the stress response behavior of plants following *msh1* developmental reprogramming. We show that *msh1* mutants display a differential response to abiotic and biotic stress, which could be partly explained by transcriptome changes. Epi‐lines, deriving from crosses of *msh1* with wild type*,* showed increased seed yield and higher tolerance to salt, freezing, and mild heat stress. Under prolonged cold stress, *msh1* mutants showed increased variation in DNA methylation, particularly in non‐CG context, and this increased CHG and CHH methylation pattern variation did not appear to influence the *msh1* crossing‐derived vigor phenotype. Taken together, the data imply that developmental phenotypes in the *msh1* mutants are caused by large‐scale gene expression changes associated with stress response, along with genome‐wide methylome repatterning. Methylome changes in non‐CG context were disproportionately affected by cold stress and were hyper‐responsive to environmental changes, whereas changes in CG context appeared to be stable and to influence plant phenotype.

## 2 MATERIALS AND METHODS

### 2.1 Plant growth conditions and PCR genotyping

The genetic background used throughout the study was* *Arabidopsis Col‐0 ecotype. For phenotypic measurements, seeds were sown into plastic pots containing Fafard germination mix with Turface MVP added. After 48–72 hr of cold stratification at 4°C in a dark chamber, pots were moved to growth chambers set at 22°C. The *msh1* T‐DNA mutant was obtained from Arabidopsis Biological Resource Center (SAIL_877_F01, stock number CS877617) and genotyped as described previously (Shao et al., 2017). Mutants #12‐29, #12‐4, and #9 are three selections from the parental T‐DNA line. Epi‐lines were developed by crossing wild type with *msh1* mutants, some of which had been exposed to cold stress (S), and subsequently self‐pollinating filial generations. PCR genotyping as previously described (Shao et al., 2017) was performed on the F_2_ population, and only plants with wild‐type *MSH1/MSH1* were evaluated and forwarded. Yield and stress tests were performed on bulked epi‐F_3_ populations or F_2:3_ lines developed from progeny of individual F_2_ plants. A Supporting information Figure S1 describes the stress treated *msh1* mutants and development of epi‐lines.

### 2.2 Abiotic and biotic stress treatments

All stress treatments were performed on wild‐type Col‐0, *msh1* mutants #9, #12‐4 and #12‐29, epiF_3_ populations derived from crosses WT x *msh1*‐N, WT x *msh1*‐VD, WT x *msh1*‐N(S), and WT x *msh1*‐VD(S) that involved the two phenotypic classes of *msh1* mutants, normal phenotype (N), and variegated dwarf (VD), with and without exposure to stress (S).

Seeds for stress treatments were bleach sterilized and sown on half‐strength MS medium containing 1.5% sucrose and 0.5% MES, pH 5.7, solidified with 4% agar in sterile plastic Petri plates. For 200 mM salt germination tests, 11.7 g of NaCl was added to the growth media before sterilization. After 48–72 hr of cold stratification in a dark room at 4°C, plates were moved to Percival growth chambers set at 22°C and 16/8 light/dark cycle. Germination was scored based on root length of more than 3 mm at 2 weeks after plates were moved to the growth chamber.

For freezing tolerance, 2‐week‐old seedlings were cold acclimatized for 1 week at 4°C in 12/12 hr light/dark photoperiod. Freezing tests were performed as previously described (Barnes, Benning, & Roston, 2016), with necessary modifications. Specifically, postfreezing plates were placed in a 4°C dark chamber for 24 hr before recovery in control growth conditions for 5–7 days. Survival was scored as plants having fully expanded green rosette leaf after recovery. The *sfr2‐3* mutant (Moellering, Muthan, & Benning, 2010), used as negative control, was a kind gift from Dr. Rebecca Roston.

Two independent *MSH1* epi‐lines for each phenotypic class of *msh1* mutant were developed, WT x *msh1*‐N1 and WT x *msh1*‐N2, created by crossing two ‐*msh1* mutants with a normal phenotype (N1, N2), and WT x *msh1*‐VD1 and WT x *msh1*‐VD2 developed from two *msh1* mutants with a variegated dwarf phenotype (VD1, VD2). Seed yield was measured as total seed weight at maturity. Floral stems of 6‐week‐old plants were tied to a wooden stake and the plant enclosed completely using Arabisifter (Lehle Seeds, SNS‐03), making a pouch‐like structure in the bottom to collect shattered seeds. All four epi‐F_3_s and wild type were grown in a completely randomized design in a growth chamber at 22°C or 32°C, 16/8‐hr light/dark cycle. Seeds were carefully harvested from each population (*n* > 18 plants) at maturity. Seeds were dried in a 37°C chamber for 48–72 hr before recording seed weights.

*Pseudomonas syringae pv. tomato DC3000* strains were grown for 24 hr at 30°C on King's B media (King, Ward, & Raney, 1954) with the appropriate antibiotics, and resuspended to an OD_600_ of 0.2 (2 × 10^8^ cells ml^−1^) in 10 mM MgCl_2_. The resuspended culture was sprayed uniformly on upper and lower surfaces of fully expanded leaves of 4‐week‐old wild type, *msh1* mutant, and *msh1*‐derived epi‐lines using a jet‐spray bottle. Treated plants were well‐watered and kept in a dark room for 5 days, followed by five to 7 days in a growth chamber at 22°C and 16/8‐hr light/dark cycle before scoring for survival.

### 2.3 RNA extraction and sequencing analysis

Four‐week‐old plants grown in 22°C were transferred to a growth chamber set at 10°C, 150 μE m^−2^ s^−1^, for 30 days. Tissue from four fully expanded rosette leaves was sampled before and after 10°C transfer with three replicates per group. For each sample, frozen tissue was ground and total RNA extracted using a standard TRIzol reagent protocol. RNA samples were then treated with DNaseI (Qiagen catalog #79254). Qiagen RNeasy Plant Mini Kit (Qiagen catalog #74904) was used to purify total RNA samples prior to RNA sequencing (RNAseq). Poly(A)‐enriched RNAseq was performed by Beijing Genomics Institute (BGI), generating at least 59.6 M paired‐end, 100‐bp reads per sample. Reads were trimmed and aligned to the Arabidopsis TAIR10 reference genome sequence with annotation from Araport11 PreRelease3 using TopHat2 (Kim et al., 2013). The DESeq2 method (Love, Huber, & Anders, 2014) was used to identify differentially expressed genes (cutoff of FDR <0.05, |log2(fold change)| ≥1, and mean FPKM ≥1). Gene Ontology (GO) enrichment analysis was performed using the DAVID database (Huang, Sherman, & Lempicki, 2009). GO terms with *p*‐value <0.05 after Benjamini‐Hochberg (Benjamini & Hochberg, 1995) correction for multiple testing were considered statistically significant in each comparison.

For transposable element (TE) family expression analysis, reads were aligned using the STAR 2‐pass method (Dobin et al., 2013), allowing up to 100 multi‐mapped locations as per the recommendation of TEtranscripts (Jin, Tam, Paniagua, & Hammell, 2015). Quantification and testing for differential expression of TEs were performed using TEtranscripts with the developer‐provided Arabidopsis TE family annotation.

### 2.4 Cold stress methylome analysis

To obtain whole‐genome bisulfite sequencing data for the cold stress experiment, plants were grown in a controlled growth chamber set to 10°C, 150 μE m^−2^ s^−1^ or 500 μE m^−2^ s^−1^, and 12/12 day/night photoperiod for 21 days, beginning from germination, then moved to recovery at 22°C, 250 μE m^−2^ s^−1^ for 18 days before sampling. Control plants were grown continuously at 22°C, 250 μE m^−2^ s^−1^ from sowing, and sampled upon reaching a similar developmental stage as cold‐stress recovered plants. Four fully expanded rosette leaves from each individual plant were harvested and DNA extracted as previously described (Li & Chory, 1998), with two replicates per group. Library generation and bisulfite‐sequencing were performed by BGI on a Hiseq2000. Reads were aligned to the TAIR10 reference genome using Bismark (Krueger & Andrews, 2011) with default mismatch parameters. Due to the potential for artifacts, cytosines of CCC context were excluded from CHH analysis. Methylation conversion rates as determined by unmethylated chloroplast DNA ranged from 98.91% to 99.29% (Supporting information Dataset S1).

The R package *methylKit* 1.1.8 (Akalin et al., 2012) was used to call DMRs, based on 100 bp nonoverlapping windows, separately for CG, CHG, and CHH contexts. Only cytosine base positions with ≥3 reads were retained for analysis, and normalized methylation counts for each cytosine were used based on coverage. Windows with ≥5 cytosines (of the given context) were considered for analysis, to rule out low information regions. The principal component analysis was performed using the *PCASamples* function. Subsequent comparison between treatment (cold or control) and genotype (*msh1* T‐DNA or wild type) combinations were performed by logistic regression with *methylKit*. DMRs for each context were identified based on a methylation difference of at least 10% absolute value and a *q*‐value <0.05, then clustered using Ward's method (Ward, 1963). For CG context, genes overlapping DMRs within each cluster were identified and subjected to GO enrichment analysis using the DAVID database (Huang et al., 2009). For CHG and CHH contexts, TE's overlapping DMRs within each cluster were identified and tested for enrichment of TE families and superfamilies (annotated by TAIR10) using the hypergeometric test (FDR < 0.01).

## 3 RESULTS

### 3.1 The *msh1* mutant shows variable abiotic and biotic stress tolerance

**Figure 1:**
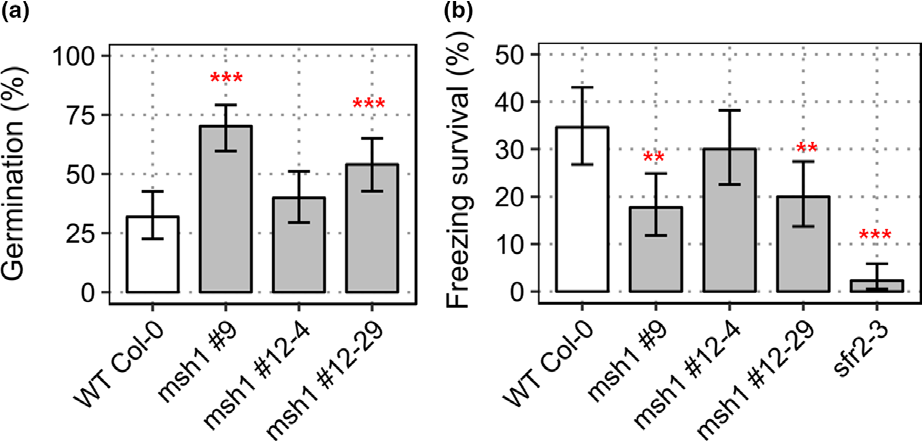
Abiotic stress tests in Arabidopsis *msh1* mutants. (a) Percent germination rate of wild type Col‐0 and*, msh1* mutants #9, #12‐4, and #12‐29 at 200 mM NaCl‐supplemented growth media, scored after 2 weeks postsowing [*n* = 100 plants each; error bars represent standard error of means (*SEM*)]. (b) Proportion of recovered (survived) plants 7 days postfreezing treatment at −10°C. *sfr2‐3* was used as negative control for freezing tolerance (*n* = 130, error bars represent *SEM*). Significance at ‘***’ 0.001 ‘**’ 0.01 ‘*’ 0.05 ‘.’ 0.1

Previous studies have shown that *msh1* mutants are more tolerant to drought, high light, and heat stress (Shedge et al., 2010; Virdi et al., 2016; Xu et al., 2011). We tested for other abiotic stress effects, focusing first on salt and freezing temperature. Seeds of *msh1* mutant and wild type were grown on plates with half‐strength MS media and 200 mM NaCl. The 200 mM NaCl concentration is highly selective for germination tests in Col‐0 (Wibowo et al., 2016). Germination was scored based on root length of greater than 3 mm at 2 weeks after sowing, assessed in three independent experiments. Only 32% percent of wild‐type seeds germinated on 200 mM NaCl plates. Two selections from the *msh1* mutants, #9 and #12‐29, showed significantly higher germination than wild type (*p*‐value 1.25 × 10^−10^ and 1.28 × 10^−4^, respectively), while *msh1*#12‐4 did not show significant difference (*p*‐value 0.148). These results suggest higher salinity tolerance in *msh1* mutants, with variation in mutant sub‐populations (Figure 1a). This result is consistent with gene expression data from *msh1* mutants (Shao et al., 2017), which show differential expression for 493 (Supporting information Dataset S2) of the 1667 salt stress–responsive genes identified through comparative microarrays (Sham et al., 2015).

To examine whether or not *msh1* mutants also showed tolerance to freezing temperatures, 2‐week‐old seedlings of wild‐type and *msh1* T‐DNA mutants were cold acclimatized for a week before exposure to −2°C for 4 hr, followed by nucleation and −10°C for 12 hr. Survival was scored as the presence of green rosette leaves 1 week after recovery under normal growth conditions (22°C, 16/8 light/dark cycle). Surprisingly, the survival rate of *msh1* mutants was lower than that of wild type (Figure 1b), indicating that the mutants are not tolerant to all stresses. Under our experimental conditions, 34.5% wild type survived −10°C. The *msh1* mutants #9 and #12‐29 showed significantly higher susceptibility to freezing temperatures (*p*‐value 0.002 and 0.009, respectively), while *msh1* mutant #12‐4 was not significantly different from wild type (*p*‐value 0.42651). From a set of 590 differentially expressed genes correlated with acclimated and nonacclimated freezing tolerance (Hannah et al., 2006), only 64 were altered in expression in the *msh1* variegated dwarf mutant (Supporting information Dataset S3).

Because *msh1* mutants have increased tolerance to abiotic stresses such as drought, heat, high light, and salt, we tested whether or not they were likewise more resistant to biotic stress. We challenged *msh1* mutants with the gram‐negative bacterial pathogen *Pseudomonas syringae* pv. *tomato* DC3000, which causes bacterial speck disease in tomato and is pathogenic to Arabidopsis. The *msh1* mutants showed susceptibility to the bacterial pathogen. While 87.5% of wild‐type plants survived the stress, *msh1* mutant sub‐populations #12‐29 and #12‐4 showed significantly higher susceptibility (Supporting information Figure S2a: *p*‐value 0.006 and 0.081, respectively). Within one population, *msh1* #9, plants with variegation and dwarfing showed significantly higher susceptibility (*p*‐value 4.35 × 10^−5^and 7.15 × 10^−7^, respectively) to *P. syringae* than *msh1* mutants with a mild phenotype (Supporting information Figure S2b: *p*‐value 0.327). Thus, *msh1* mutants are susceptible to biotic stress despite markedly increased expression of biotic stress–responsive pathways in *msh1* mutants (Shao et al., 2017), and the biotic stress response appears related to the severity of the *msh1* phenotype.

To investigate the inheritance of these stress responses following crossing between *msh1* mutants (T‐DNA) and wild type, seed germination rate in 200 mM NaCl concentration and survival of seedlings at −10°C freezing temperatures were assayed in three independent experiments for *msh1*‐derived epi‐lines in the F_3_ generation, with wild type as a control. Epi‐lines were created by crossing wild‐type Col‐0 with *msh1* mutants as pollen donor, and self‐pollinating filial generations after PCR genotyping for *MSH1/MSH1* in F_2_s to obtain epi‐F_3_ bulks (Supporting information Figure S1). When seeds were germinated on plates with 200 mM NaCl, WT x *msh1*‐N, and WT x *msh1*‐VD showed significantly higher germination rate (*p*‐value 7.15 × 10^−14^ and 3.20 × 10^−12^, respectively) than wild type (Supporting information Figure S3a). This result was consistent with the parental *msh1* mutant, which showed a similar increase in salt tolerance (Figure 1a). However, epiF_3_ population WT x *msh1*‐VD also showed higher tolerance to freezing (Supporting information Figure S3b: *p*‐value 0.015), where *msh1* mutant showed greater susceptibility. These observations are consistent with the expectation that *msh1* x wild‐type crosses produce a different epigenetic state, thus resulting in distinctive phenotypes.

**Figure 2:**
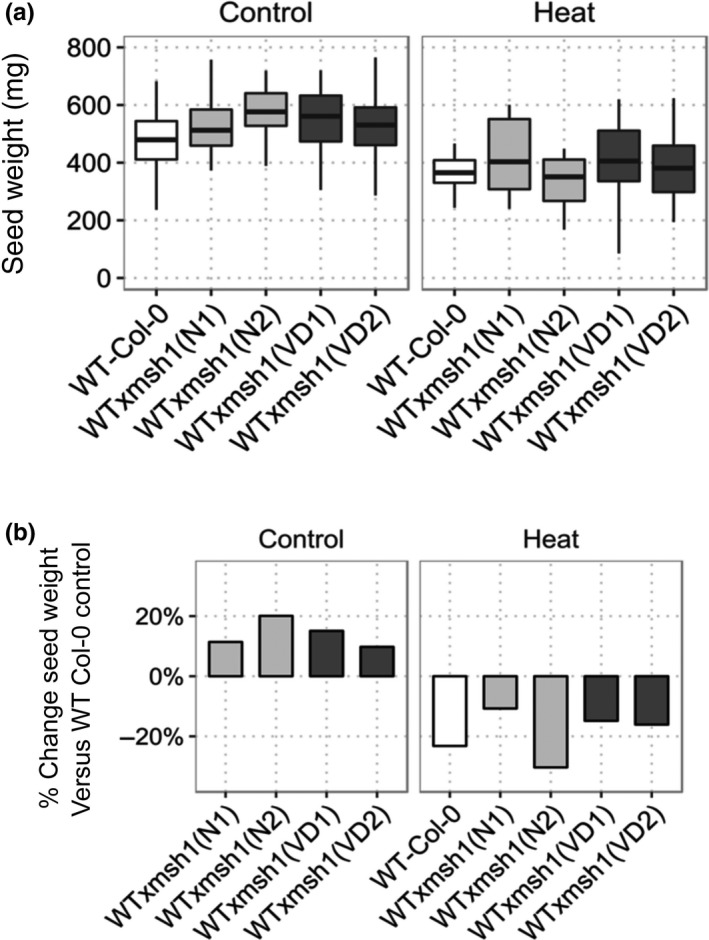
Total yield measurements for *msh‐*derived epi‐lines compared to wild‐type Col‐0. (a) Whisker plots showing differences in seed yield between wild type and *msh1* epi‐lines under control (22°C) and mild heat‐stress (32°C) growth conditions [Control (*n* = 36), heat stress (*n* = 18)]. (b) Percent change in seed weight for epi‐lines under control and heat stress condition compared to seed weight of wild type under control growth conditions

To evaluate the response of progeny from crossing under less severe, nonlethal stress, we subjected epi‐F_3_ plants to mild heat stress and measured total seed weight at harvest. For this experiment, four epi‐lines and wild type were grown in growth chambers under control (22°C) or mild heat stress (32°C) throughout the plant life cycle. Epi‐lines showed 9.7%–19.6% increase in seed yield compared to wild type in control conditions (Figure 2a). Three of the epi‐lines also performed significantly better than wild type under mild heat stress, showing 9.5%–16.5% increase in yield (Figure 2a). The lower yield penalty under mild heat stress in the three epi‐lines, coupled with the enhanced salt and cold tolerance, provides an indicator of greater yield stability and lower environmental effects on the *MSH1* growth‐enhanced phenotype (Figure 2b). These results resemble the higher yield stability observed in soybean *MSH1* epi‐lines grown across four different locations in Nebraska (Raju et al., 2018).

### 3.2 The methylome of *msh1* is hyper‐responsive to cold stress with disproportionately higher CHH hypomethylation

Transcriptome studies of *msh1* showed clear enrichment of biotic and abiotic stress response genes, including response to cold. Despite changes in cold‐responsive transcription factors (Shao et al., 2017), *msh1* mutants showed susceptibility to freezing temperatures. These observations led us to test whether *msh1* mutants would show differential methylome and transcriptome response to low‐temperature stress.

To evaluate the extent of DNA methylation changes related to long‐term cold stress, *msh1* mutants and wild‐type plants were grown at 10°C for 18 days under 12/12 light/dark cycle, then allowed to recover at 22°C for 18 days before sampling for DNA extraction. Plants were allowed to recover prior to sampling for two reasons: Plant growth was slower under cold stress, complicating the collection of sufficient tissue for methylome sequencing. In addition, we wanted to avoid transient methylation changes present during plant exposure to cold treatment.

**Figure 3:**
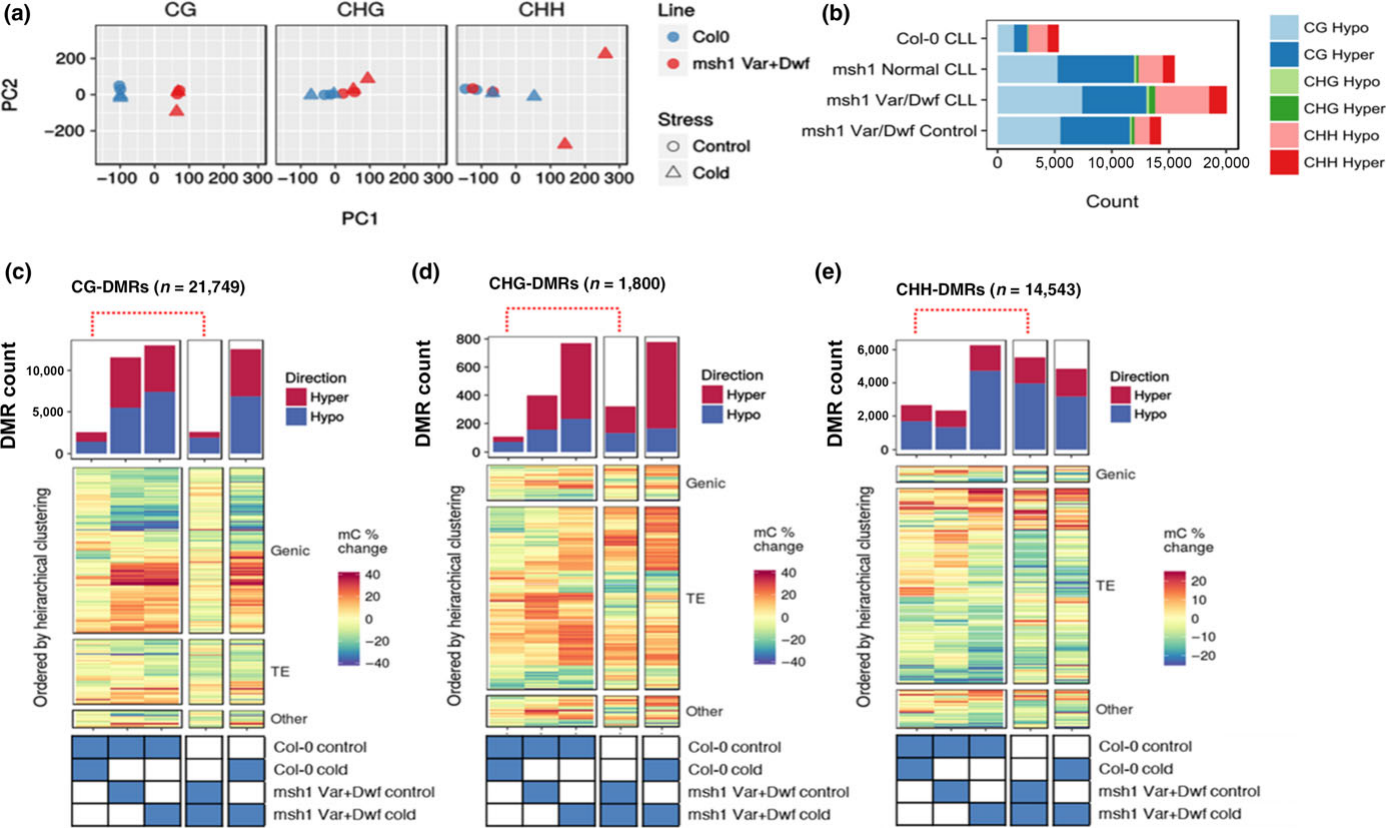
Methylome changes in *msh1* mutants and wild‐type Col‐0 from long‐term cold stress. (a) Principal component analysis (PCA) plots for methylation levels within 100‐bp windows separated for nucleotide context; CG, CHG, and CHH (H represents A, C, or T). (b) Graph of total DMR numbers in each comparison, showing hyper and hypomethylation in all three contexts. (c–e) DMR counts and hierarchical clustering of all pairwise comparisons, for CG (c), CHG (d), and CHH (e) contexts. Red dotted lines highlight *msh1* cold response relative to wild type under cold stress

To facilitate comparison of each region between different genotype and treatment combinations, methylome analysis was performed using fixed 100‐bp nonoverlapping windows. Principal component analysis plots from the first two principal components using the upper 0.9 quantile of variable windows showed CG methylation separating by genotype between wild type and *msh1*mutants, with or without stress (Figure 3a). These observations are consistent with studies of the *msh1* memory lines, where CG methylation is predominant in association with a memory phenotype (Sanchez et al., 2018). CHG methylation showed a similar pattern, although cold‐stressed samples were discriminated from control samples in *msh1* mutants more than in wild type (Figure 3a). Notably, CHH methylation showed the greatest degree of discrimination for the cold stress treatment, predominantly in *msh1* mutants (Figure 3a). Together, these results indicate that cold stress influences DNA methylation in all methylation contexts, but there is evidence of interaction with the *msh1* background, amplifying the effect in CHH context.

### 3.3 Genomewide distribution of DMRs in wild type and *msh1* mutants in response to cold stress

We investigated the number and genomic distribution of differentially methylated regions (DMRs). DMR calling was based on logistic regression over 100 bp nonoverlapping window, using a threshold of more than 10% absolute change in methylation level in each cytosine context. The resulting number of DMRs (Table 1, Figure 3b) confirmed trends observed by principal component analysis (Figure 3a). As expected, CG‐DMRs mostly occurred over genes and were relatively few between cold and control treatments in wild type or *msh1* while comparing any *msh1*group to wild type (Supporting information Figure S4a). We found 11,579 CG‐DMRs, 399 CHG‐DMRs, and 2332 CHH‐DMRs when comparing *msh1* to wild type under control conditions. Almost equal numbers of DMRs were hyper or hypomethylated in symmetric methylation context, while in CHH context, there were 30% more hypomethylated DMRs in *msh1* (Table 1, Figure 3b). We also detected 2,592 CG‐DMRs, 109 CHG‐DMRs, and 2658 CHH‐DMRs induced by cold stress alone in the wild type. The magnitude of CG and CHG changes in the *msh1* mutant was four times higher than changes induced by cold stress alone in wild type, while the magnitude of CHH changes was similar. This implies that relative to CG and CHG methylation, cold stress disproportionately affects CHH methylation, consistent with previous reports of methylome behavior under low temperature (Dubin et al., 2015).

**Table 1:** Number of DMRs in all three cytosine contexts across multiple comparisons

Comparison	Direction	CG	CHG	CHH
*msh1‐VD* vs WT	Hyper	6,085	240	996
	Hypo	5,494	159	1,336
*msh1‐VD* (S) vs *msh1‐VD*	Hyper	700	186	1,574
	Hypo	1,926	135	3,965
WT(S) vs WT	Hyper	1,154	37	977
	Hypo	1,438	72	1,681
*msh1‐VD* (S) vs WT	Hyper	5,626	538	1,548
	Hypo	7,400	232	4,723
*msh1‐VD* (S) vs WT(S)	Hyper	5,707	611	1,675
	Hypo	6,829	167	3,176

We examined whether *msh1* background affects methylation changes upon cold stress. We found 2626 CG‐DMRs, 321 CHG‐DMRs, and 5539 CHH‐DMRs between stressed and unstressed *msh1* mutant (Table 1, Figure 3b). Thus, CHH methylation, primarily over transposable elements (Supporting information Figure S4a), showed the greatest effect of cold treatment within the *msh1* background, consistent with separation seen in the PCA plot (Figure 3a). Although CHH methylation is affected by cold stress in wild type, CHH DMRs in cold‐stressed *msh1* are twice as abundant as in similar wild type comparisons. Whereas CG DMR patterns were nearly identical for cold‐stressed and control *msh1* mutants when compared to wild type, CHG and CHH DMRs showed a clear distinction in patterns, with several loci switching between hyper and hypomethylation (Figure 3c–e). These results indicate an interaction between the *msh1*effect and cold stress, such that non‐CG methylation patterns are disproportionately affected.

### 3.4 Non‐CG methylome changes in association with transposable elements

To investigate the genomic distribution of non‐CG changes in response to stress, we clustered non‐CG DMRs and looked for enrichment of TE superfamilies in these clusters. Both CHG‐ and CHH‐DMRs formed 4 clusters each (Supporting information Dataset S4). While all four clusters in CHG‐DMRs showed enrichment for DNA/En‐spm, LTR/COPIA, and LTR/Gypsy elements, clusters three (hyper) and one (hypo), which showed similar trends in all comparisons, were also enriched in LINE/L1 elements. In CHH‐DMR clusters, DNA/MuDR elements were enriched in all clusters. Cluster one, which contained the most DMRs and hypomethylation in all three comparisons (wild‐type stressed vs wild type, *msh1* vs wild type, and *msh1*‐stressed vs wild type) showed enrichment for LINE/L1, LTR/COPIA, and LTR/Gypsy elements. Clusters three and four, which showed hypermethylation in *msh1*‐stressed versus wild type, showed over‐representation of DNA/Mariner and RC/Helitron elements.

Genomic distribution of DMRs matched with known behaviors within each cytosine context. CG‐DMRs between *msh1* mutants and wild type were distributed evenly across the chromosome (Supporting information Figure S5b,c,e), while CG DMRs from cold stress were primarily limited to heterochromatin (Supporting information Figure S5a,d). CHG‐DMRs and CHH‐DMRs were mainly in heterochromatic regions for both comparisons. This finding is consistent with previous reports of cold stress methylome changes showing heterochromatin bias (Dubin et al., 2015). We examined expression changes in genes related to DNA methylation machinery. Interestingly, CHROMO METHYLTRANSFERASE 3 (CMT3) and DECREASE IN DNA METHYLATION 1 (DDM1) expression were down‐regulated in cold‐stressed *msh1* mutants compared to unstressed mutants and wild type (Supporting information Figure S6). As *cmt3* and *ddm1* mutants are known to increase heterochromatic TE de‐repression, these observations appear consistent with CHH hypomethylation of heterochromatin in the interaction of *msh1* effect and low‐temperature stress.

### 3.5 Transcriptome response of Arabidopsis *msh1* mutants under chronic cold stress

**Figure 4:**
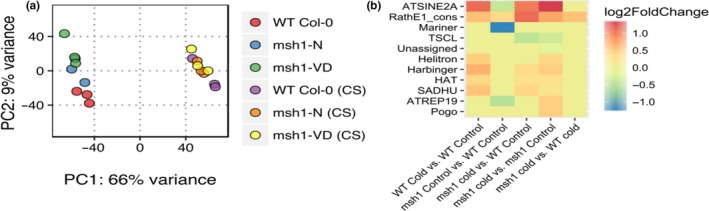
Transcriptome changes in *msh1* mutants before and after chronic cold stress. (a) PCA plot from normalized log values of gene expression from wild‐type and *msh1* mutants under control or cold stress. (b) Heat map showing differential expression of transposable element (TE) super families from each corresponding comparison within cold stress experiment

We evaluated the effect of cold stress on the transcriptome of *msh1* mutants. Wild‐type Col‐0 and *msh1* plants were grown at 22°C for 4 weeks before leaf tissue was collected (control group), or grown at 10°C for an additional 30 days before sampling tissues for RNA extraction (cold‐stressed group). Transcriptome analysis showed cold stress to be the largest contributor to transcriptional changes within the experimental groups, evident from the groups formed in PCA plotting with normalized log values of gene expression (Figure 4a). Although the magnitude of gene expression change was lower than transcriptome change in our earlier report (Shao et al., 2017), similar pathways were modulated in both *msh1* mutants with or without severe phenotype, including defense, jasmonic acid, abiotic stress response, photosynthesis, and oxidative stress (Supporting information Dataset S5). Technical differences, like differential developmental staging and changes in circadian phase (Hsu & Harmer, 2012), might explain the differences in the magnitude of transcriptome changes. Pathways affected in *msh1* appear to be induced by cold alone in wild type, suggesting that *msh1* mutants have stress response pathways activated in the absence of any environmental cues. Response to abiotic stress (cold, salt, light, and wounding) and biotic stress (response to chitin and jasmonic acid) is activated as a cold stress response in wild type and is also activated in *msh1* (Supporting information Figure S7a). Defense response, jasmonic acid‐mediated signaling, and photosynthesis‐related genes were specifically enriched in *msh1* (Supporting information Figure S7a: Dataset S5). Taken together, these results suggest that unlike methylome, transcriptome changes do not show increased plasticity in *msh1* mutants under cold stress.

We also looked into differences in transposable element expression using TEtranscripts (Jin et al., 2015). Mariner, ATREP19, and SINE TE superfamilies were significantly down‐regulated in *msh1* mutants compared to wild type, while Rath elements were up‐regulated in all comparisons (Figure 4b). In contrast, SINE elements showed clear cold‐stress induced up‐regulation. Similarly, Helitron, Harbinger, HAT, and SADHU elements were up‐regulated in wild type under cold stress (Figure 4b). At the family level, ATCOPIA28 and ATCOPIA31A showed clear stress‐induced up‐regulation, while VANDAL5A, ATREP3, ATCOPIA44, ATCOPIA 78, ATCOPIA 93, and ATMU1 showed down‐regulation in *msh1* mutants (Supporting information Figure S7b).

### 3.6 *MSH1*‐induced CG methylation changes are associated with enhanced growth in progeny from *msh1* crossing

**Figure 5:**
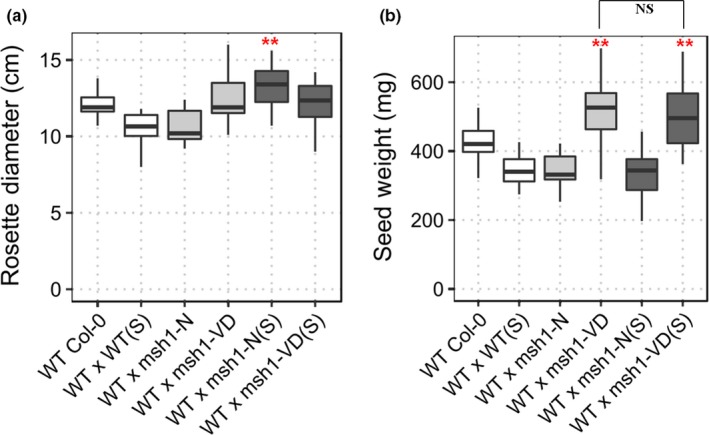
Variation in rosette diameter and seed weight in *MSH1* epi‐F_2_ population. (a) Whisker plot showing variation in rosette diameter measured at 6 weeks after sowing (*n* = 18). (b) Whisker plot showing variation in total seed weight measured carefully after bagging the plants with Arabisifter (Lehle Seeds, SNS‐03). Epi‐F_2_ populations were developed from cold stressed (S) and unstressed *msh1* mutants with (VD) or without (N) phenotype. F_2_ plants were selected after genotyping for *MSH1/MSH1* wild‐type allele. Rosette diameter and seed weight were measured from the same set of F_2_ plants. Significance at ‘***’ 0.001 ‘**’ 0.01 ‘*’ 0.05 ‘.’ 0.1

To evaluate the extent to which non‐CG methylome divergences affect the *msh1* crossing‐derived enhanced growth phenotype in Arabidopsis (Virdi et al., 2015), we investigated the epi‐lines from *msh1* mutants with or without cold stress (see Methods). We assayed rosette diameter, days to flowering, and total seed weight from F_2_ and selected F_2:3_ lines (progeny from selected individual F_2_ plants) under control growth conditions. The F_2_ population WT x *msh1*‐N(S) showed higher mean rosette diameter compared to wild type, measured at 6 weeks after sowing (Wilcox test, padj 0.004, Figure 5a). This population flowered an average of 2 days earlier (Wilcox test, padj 0.002, Supporting information Figure S8). Both WT x WT(S) and WT x *msh1*‐N populations showed smaller rosette diameter compared to wild type (Figure 5a). Mean seed yield, measured in milligrams, for WT x *msh1*‐VD(S) and WT x *msh1*‐VD was significantly higher than wild‐type Col‐0 (Wilcox test, padj 0.015, Figure 5b), whereas no significant difference was found between WT x *msh1*‐VD(S) and WT x *msh1*‐VD (*t* test, *p*‐value 0.80). These results show that for epiF_2_s, WT x *msh1*‐VD(S) and WT x *msh1*‐VD showed 20% and 17.9% increase in yield compared to wild type, but stressing the *msh1* mutant prior to crossing does not have a significant effect on yield. We also noticed that while WT x *msh1*‐VD and WT x *msh1*‐VD(S) showed increases in seed yield, WT x *msh1*‐N(S) showed higher rosette diameter compared to wild type, suggesting the possibility of selection for separate traits of vegetative biomass heterosis and seed yield heterosis in *msh1* derived epi‐lines.

**Figure 6:**
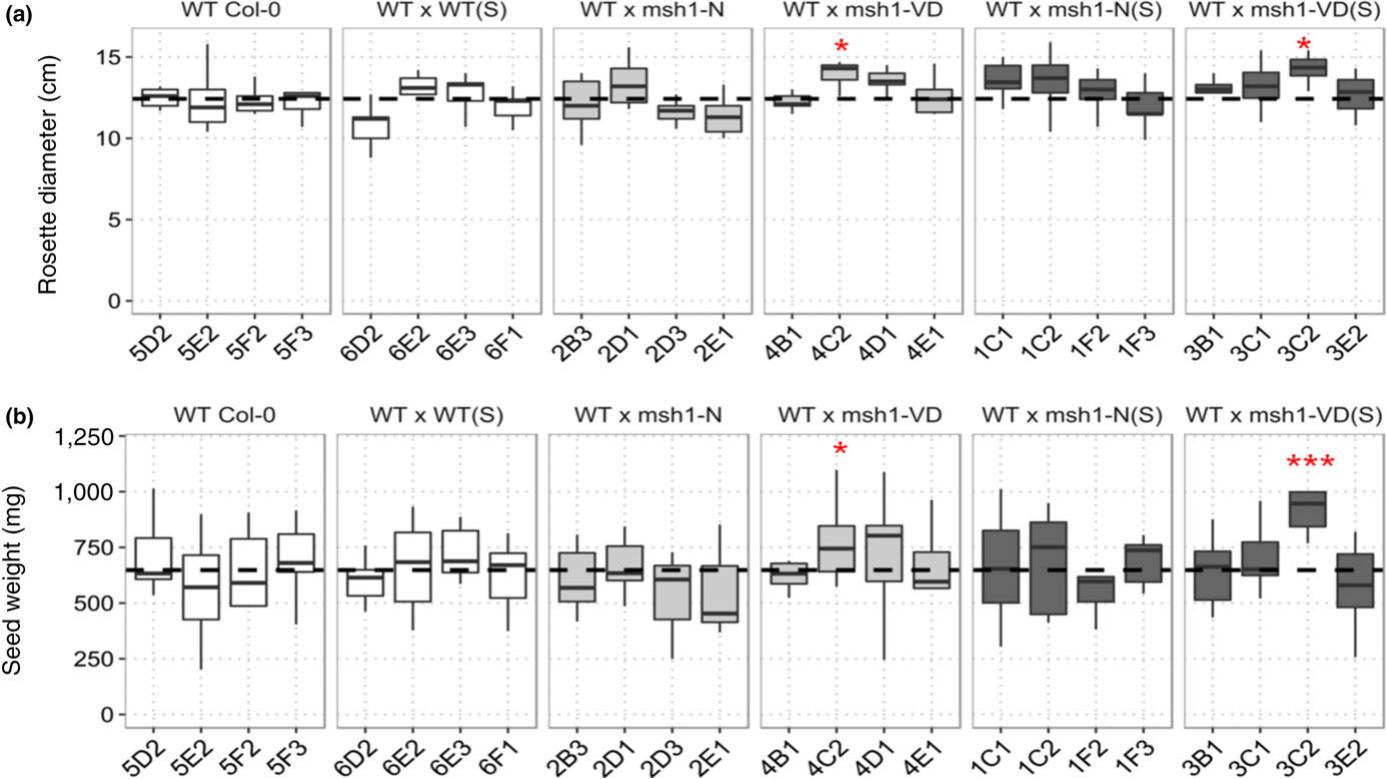
Rosette diameter and total seed weight in selected epiF_2:3_. (a) Seed weight measurements from top 20% selection in each epi‐F_2_ population, including four sub‐lines for wild type and WT x WT(S). Seed weight was measured in milligrams dried seeds collected from plants bagged with Arabisifter (Lehle Seeds, SNS‐03). Black dotted line represents wild‐type average (*n *=* *9 plants each). (b) Rosette diameter measured from the same plants at 6 weeks postsowing. Significance at ‘***’ 0.001 ‘**’ 0.01 ‘*’ 0.05 ‘.’ 0.1

We evaluated total seed weight for F_2:3_ lines following selection of the upper 20% for seed weight in each F_2_ population under control conditions. Although the selection was performed on seed weight, F_2:3_ epi‐lines 3C2, derived from WT x *msh1*‐VD(S), and 4C2, derived from WT x *msh1*‐VD, showed significantly higher rosette diameter (Wilcox test, padj = 0.018, Figure 6a) compared to wild type. Both epi‐lines also showed significantly higher seed weight compared to average wild type (*t* test, *p*‐value = 0.0008 and 0.043 respectively, Figure 6b), reflecting a response to selection. Similar to F_2_ results, there was no significant difference in seed weight between 3C2 and 4C2 (*t* test, *p*‐value = 0.203), confirming that stress treatment of *msh1* mutants does not have an effect on *msh1*‐derived growth enhancement in progeny from crosses.

**Figure 7:**
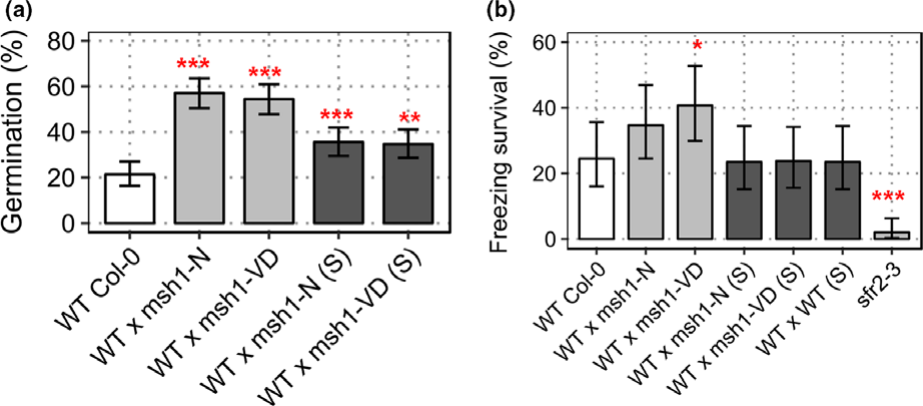
Abiotic stress tolerance in epi‐F_3_s derived from cold‐stressed (S) and unstressed *msh1* mutants. (a) Percent germination of wild‐type Col‐0 and epi‐F_3_ bulks; WT x *msh1*‐N, WT x *msh1*‐VD, WT x *msh1*‐N(S), WT x *msh1*‐VD(S) at 200 mM NaCl‐supplemented growth media. Each bar represents three replicates (*n *=* *225), and error bars show SEM. (b) Percent survival of wild type and epi‐F_3_s, after 12 hr at −10°C. *sfr2‐3* was used as negative control. Survival was scored after 1 week recovery from three replicates (*n *=* *100), and error bars represent *SEM*

We subsequently tested whether or not observed methylome changes had an effect on stress adaptation of derived epi‐lines. Surprisingly, epi‐lines derived from the cold‐stressed *msh1* mutant as parent showed a different response to salt and freezing stress. Whereas WT x *msh1*‐VD(S) and WT x *msh1*‐N(S) epi‐F_3_ populations were significantly more tolerant to salt stress (*p*‐value 9.1 × 10^−4^ and 1.8 × 10^−3^, respectively), although lesser in magnitude to comparable populations from unstressed (Figure 7a), they were not significantly different from wild type in their response to freezing stress (*p*‐value 0.867 and 0.904, respectively, Figure 7b). These results suggest that an interaction exists between *msh1* effect and cold stress effects. Taken together, data indicate that stressing *msh1* mutants triggers a disproportionate increase in non‐CG methylation, but these changes do not affect *msh1*‐derived growth vigor and can negatively affect stress adaptation in *msh1*‐derived epi‐lines.

## 4 DISCUSSION

Previous studies have shown *msh1* mutants to be more tolerant to high light, drought, and heat stress (Shedge et al., 2010; Virdi et al., 2016; Xu et al., 2011), consistent with enrichment for abiotic stress response genes (Shao et al., 2017). While we saw increased tolerance for salt stress, *msh1* mutants showed lower survival rate at freezing temperature and in response to the bacterial pathogen *P. syringae*. The seeming incongruity between activation of multiple stress pathways and susceptibility to freezing temperatures may be due to specific mechanisms underlying freezing tolerance in plants, which include plastid membrane remodeling (Moellering et al., 2010). Indeed, low frequency, localized plastid genome changes are reported in *msh1* mutants, along with a reduction in the number of plastids per cell and dramatically altered thylakoid membrane structure in a portion of the organelle population (Xu et al., 2011). Also, loss of *MSH1* might affect the functions of its putative protein interactors, such as the PsbP family protein PPD3 (Virdi et al., 2016), which could further impact the plastid. Alternatively, *msh1* mutants may be unable to mount an appropriate response to freezing stress due to desynchronization of the circadian clock (Shao et al., 2017), which influences freezing tolerance (Maibam et al., 2013). Freezing tolerance is impaired in *cca1‐11/lhy‐21* double mutants, and *gi‐3* mutants are susceptible to freezing stress due to impaired sugar metabolism (Cao, Ye, & Jiang, 2005; Dong, Farré, & Thomashow, 2011). Also, CBF1 and CBF3 genes, which are positive regulators of cold acclimatization (Novillo, Medina, & Salinas, 2007), are down‐regulated in *msh1* mutants (Shao et al., 2017).

A recent study has suggested that miR163 is a negative regulator of defense response to *P. syringae* in Arabidopsis (Chow & Ng, 2017). Interestingly, *msh1* mutants with variegation and dwarfing showed up‐regulation of miR163, while *msh1* mutants with subtle mutant phenotype did not show any changes (Shao et al., 2017). The up‐regulation of miR163 in *msh1* mutants with pronounced phenotype corresponds well with their susceptibility to the bacterial pathogen, while mutants with mild *msh1* phenotype show survival rates similar to wild type (Supporting information Figure S2b). Therefore, one possible explanation for observed stress responses is that organellar changes and/or modulation of key regulatory genes might affect particular stress response, while the vast majority of transcriptional changes may comprise a compensatory response that does not affect the phenotypic outcome.

Whole‐genome bisulfite sequencing of *msh1* mutants previously revealed numerous changes in DNA methylation over both genic regions and transposable elements (Virdi et al., 2015), raising the possibility of epigenetic feedback as a response to *MSH1* loss, and heritable methylation changes at stress‐responsive loci (Kinoshita & Seki, 2014). Enhanced tolerance to salt stress in epi‐lines developed by crossing wild‐type Col‐0 with *msh1* mutants supports the heritability of methylation changes at stress–responsive loci. The derived epi‐lines also showed tolerance to freezing stress, despite the parental *msh1* mutant showing susceptibility to freezing temperatures. It is possible that circadian regulation may resynchronize following the crossing of *msh1* mutants with wild type, which is known to influence freezing tolerance in plants (Maibam et al., 2013). One potential limitation of this study is that we did not sequence the methylomes of epi‐lines derived from various *msh1* crosses, which could have shed more light on the regulation of genes responsible for differences in stress tolerance and growth behavior. In comparable soybean epi‐F_4_ lines, circadian genes *GI* and *PRR3/5/7* were up‐regulated (Raju et al., 2018), suggesting that modulation of circadian regulators follows crosses with *msh1*. Derived epi‐lines in soybean multi‐location field trials display higher yield stability through reduced epitype‐by‐environment effect (Raju et al., 2018). In Arabidopsis, we likewise observed a lower yield penalty under mild heat stress in epi‐lines compared to wild type (Figure 2b), implying higher buffering across environments. These observations invite more detailed investigation of the link between *msh1* derived epigenetic variation and decreased environment interaction in derived epi‐lines.

Long‐term cold stress disproportionately affects *msh1* mutants, which show an amplified CHH hypomethylation response primarily in the heterochromatic region. It is notable that epigenetic changes reported in *msh1* mutants under cold stress mainly involve non‐CG methylation at TE sites, predominantly retroelements known to be affected by stress (Wessler, 1996). We also see down‐regulation of *CMT3* and *DDM1* in cold‐stressed *msh1* mutant compared to wild type and unstressed *msh1* (Supporting information Figure S6), which is implicated in heterochromatic TE derepression. The *msh1* mutants showed significant differences in expression of TE superfamilies. Differentially expressed TEs belonged to Rath elements, SINEs, and Mariner superfamilies known to contain shorter TEs, on average (Lewsey et al., 2016), that are usually methylated by the DRM1/2 pathway (Stroud et al., 2013, 2014). Mariner TE sequences are significantly underrepresented in exons and are often absent in GC‐rich genic regions of the genome (Lockton & Gaut, 2009).

Transcriptome studies showed that stress was consistently the major contributor to gene expression changes in wild type and *msh1* mutants. This is expected as changes in CHG and CHH methylation in Arabidopsis are concentrated around the pericentromere, while CG changes are distributed throughout the genome, and non‐CG methylation changes are unlikely to direct gene expression changes. A recent study involving multiple ecotypes in Arabidopsis has shown CHH hypomethylation from lower temperatures, with much of the temperature variation in CHH methylation due to components of the RdDM pathway (Dubin et al., 2015). Reports of chromatin changes and epigenetic features of stress memory in plants and observations that some epigenetic mutants have altered stress responses support the argument that these changes may have biological roles (Probst & Mittelsten Scheid, 2015). Interestingly, the increased variation in non‐CG methylome divergence in *msh1* mutants does not seem to have any significant effect on the previously described *msh1*‐derived enhanced growth phenotype (Virdi et al., 2015), emphasizing the importance of *MSH1*‐induced CG methylation changes in this phenomenon. CG methylation changes are more stably transmitted to progeny than non‐CG changes (Saze, Scheid, & Paszkowski, 2003). A recent study also showed that nonrepetitive sequences and higher CG content predispose a region for the transgenerational stability of inherited epigenetic features (Catoni et al., 2017). Moreover, stress‐induced epigenetic memory is conditionally heritable through the female germline (Wibowo et al., 2016). This excludes the possibility of inheritance of stress‐induced methylation changes, particularly non‐CG changes to the crossed progeny of this study since *msh1* mutants were used as the pollen donor.

Results from this study indicate that *msh1* methylomes are hyper‐responsive to environmental stress in a manner distinct from the wild‐type response, and identification of the *msh1* background as a modifier of cold‐induced CHH hypomethylation provides an experimental system to further understand mechanisms that control temperature‐responsive methylation changes and their inheritance behavior in crossed and selfed progeny. The experimental results indicate that CG methylation changes rather than non‐CG in *msh1* mutants as an influence on growth behavior of epi‐lines following crossing with wild type.

## DATA AVAILABILITY

Raw sequence data from the methylome and transcriptome is available for download at NCBI Sequence Read Archive under the BioProject ID: PRJNA482906 and PRJNA482911 respectively.
