# Supplementary figures and images for: Epigenomic plasticity of Arabidopsis msh1 mutants under prolonged cold stress
Source: Plant Direct. 2018 Aug 29;2(8):e00079. doi: 10.1002/pld3.79 (PMC6508824; doi:10.1002/pld3.79)

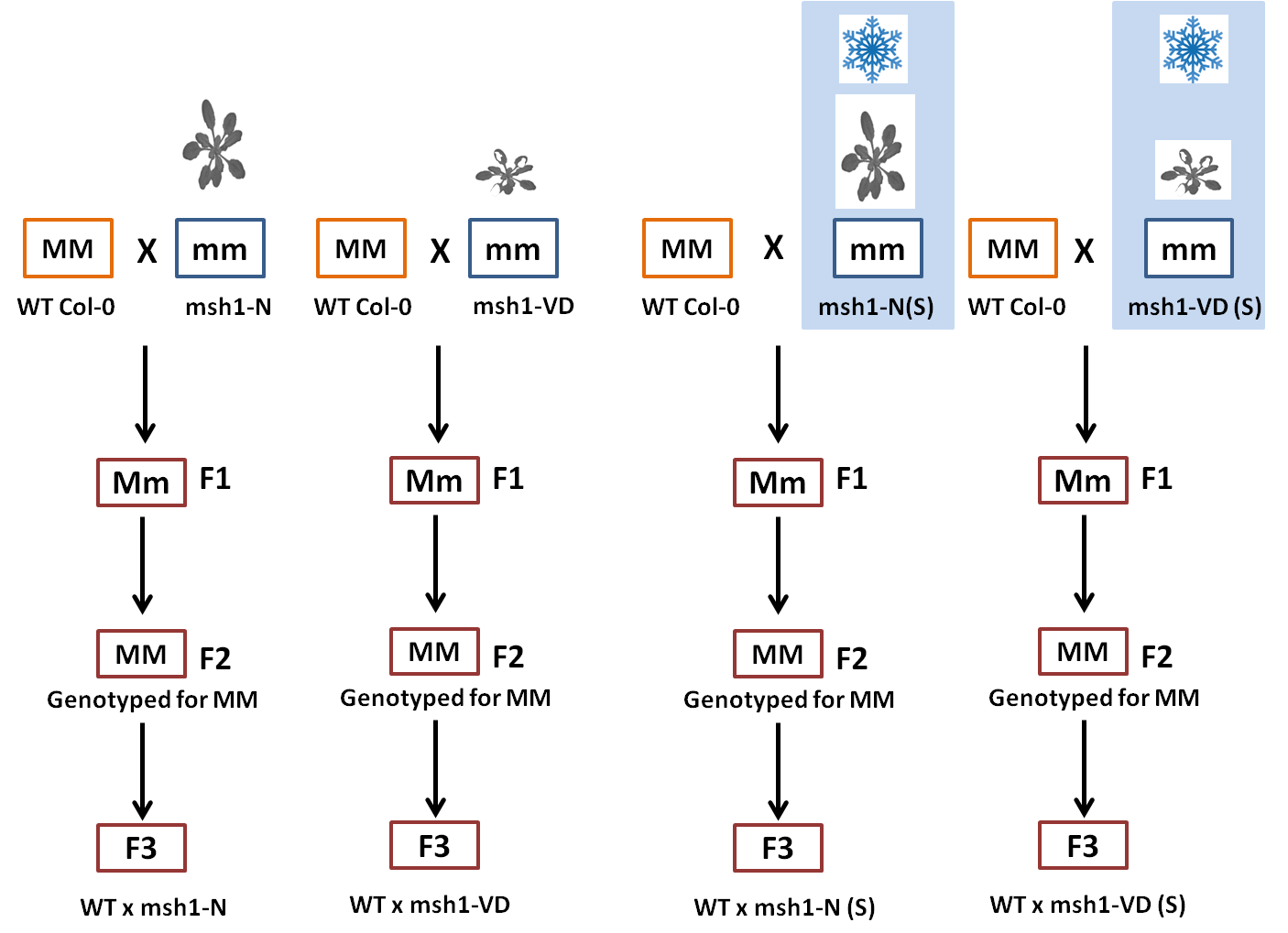

Supplement: Supplementary file 1 [file PLD3-2-e00079-s001.png]

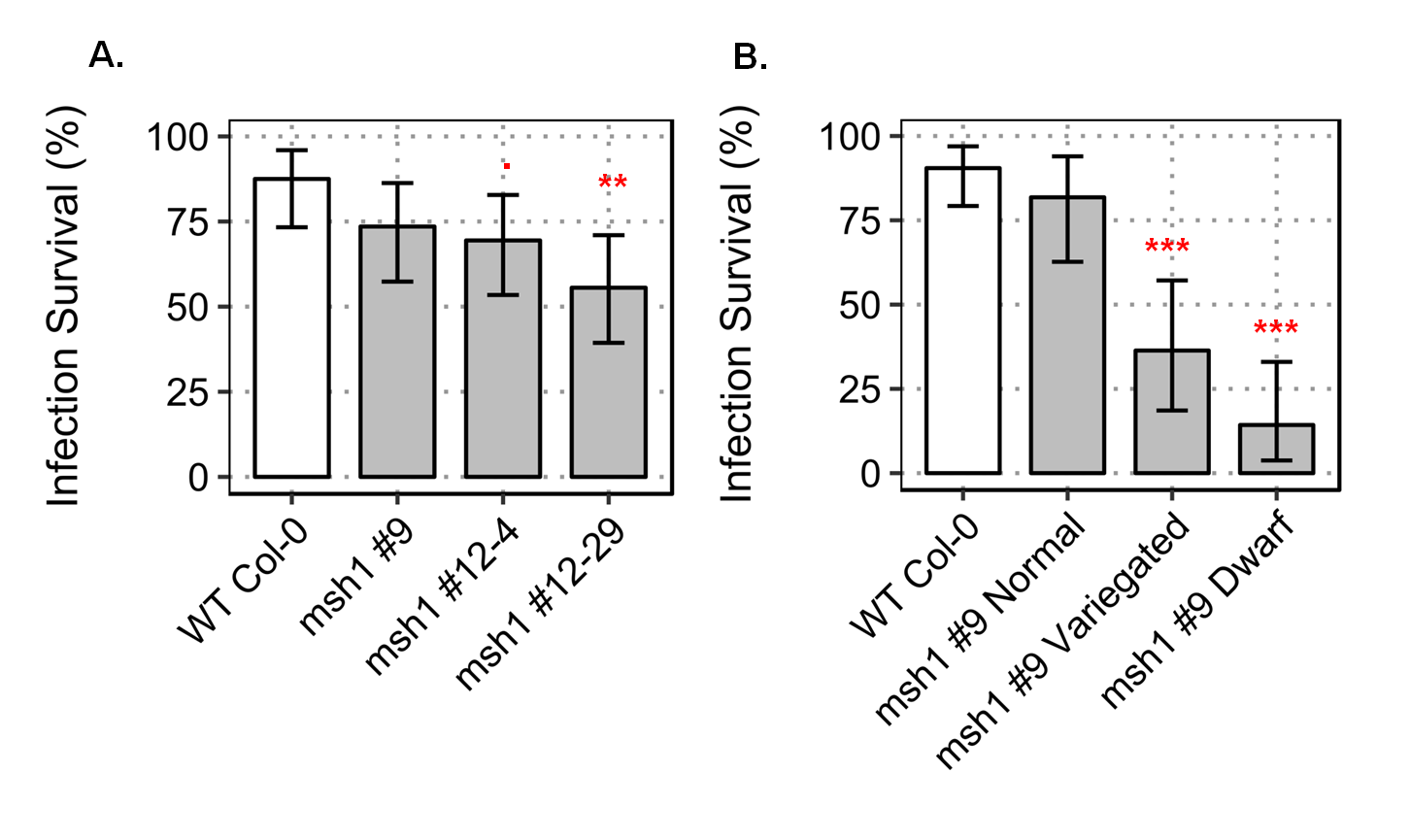

Supplement: Supplementary file 2 [file PLD3-2-e00079-s002.png]

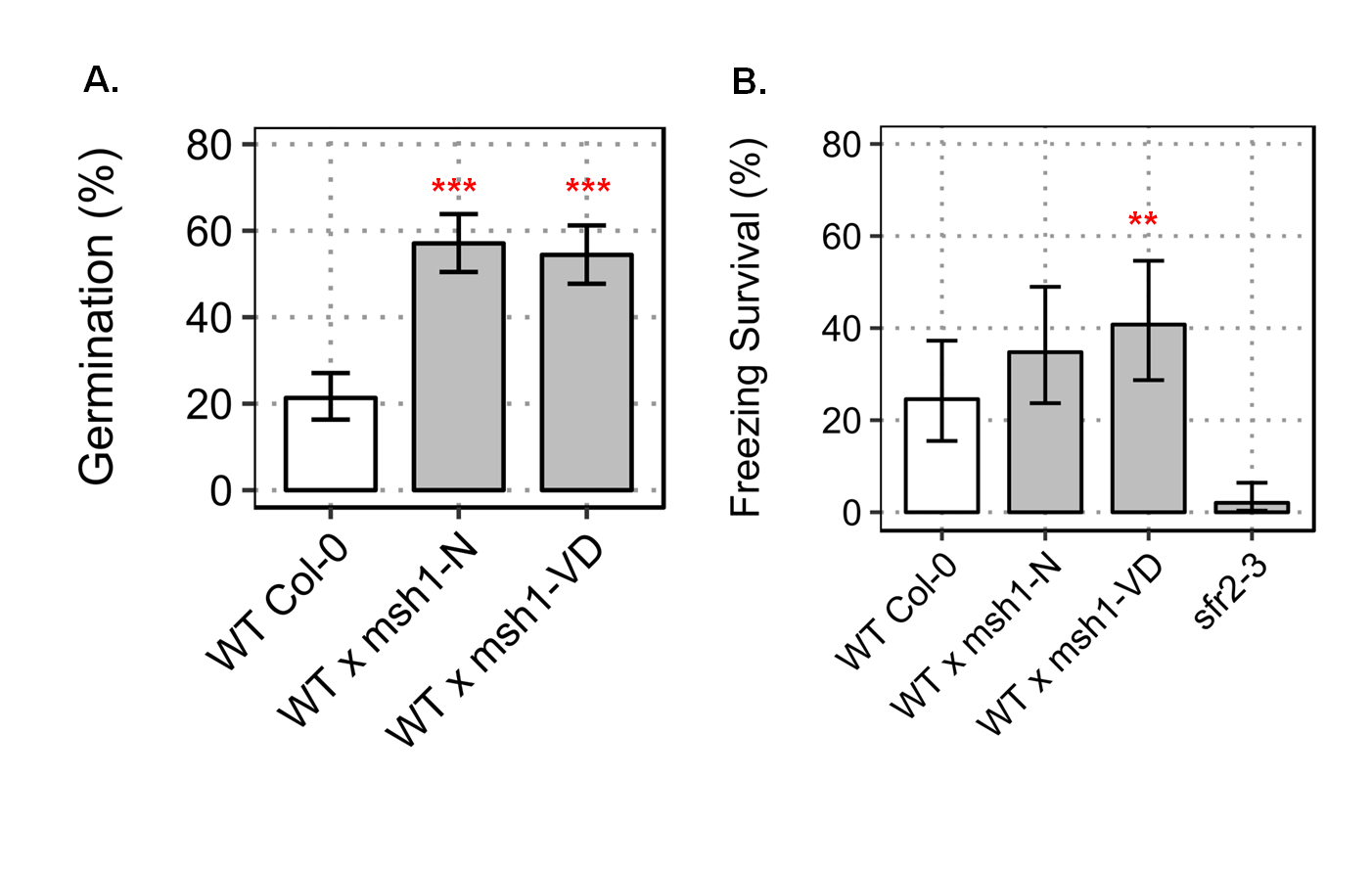

Supplement: Supplementary file 3 [file PLD3-2-e00079-s003.png]

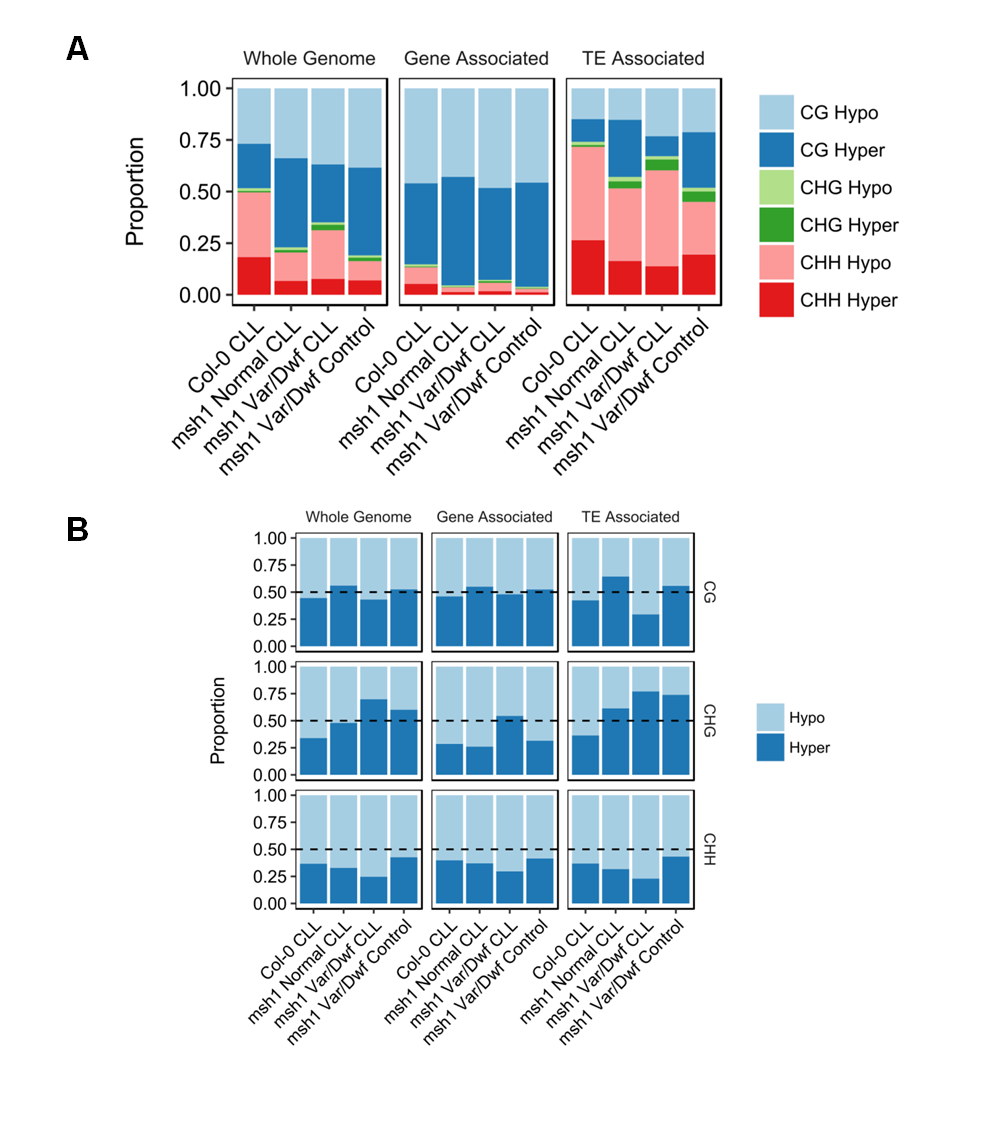

Supplement: Supplementary file 4 [file PLD3-2-e00079-s004.png]

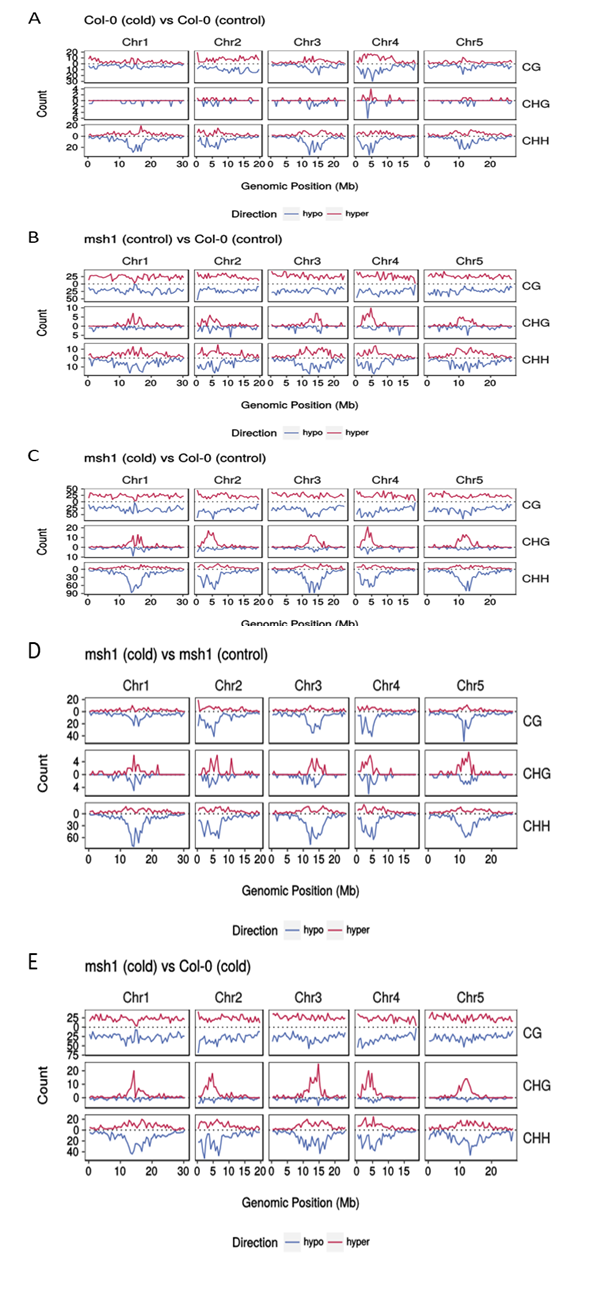

Supplement: Supplementary file 5 [file PLD3-2-e00079-s005.png]

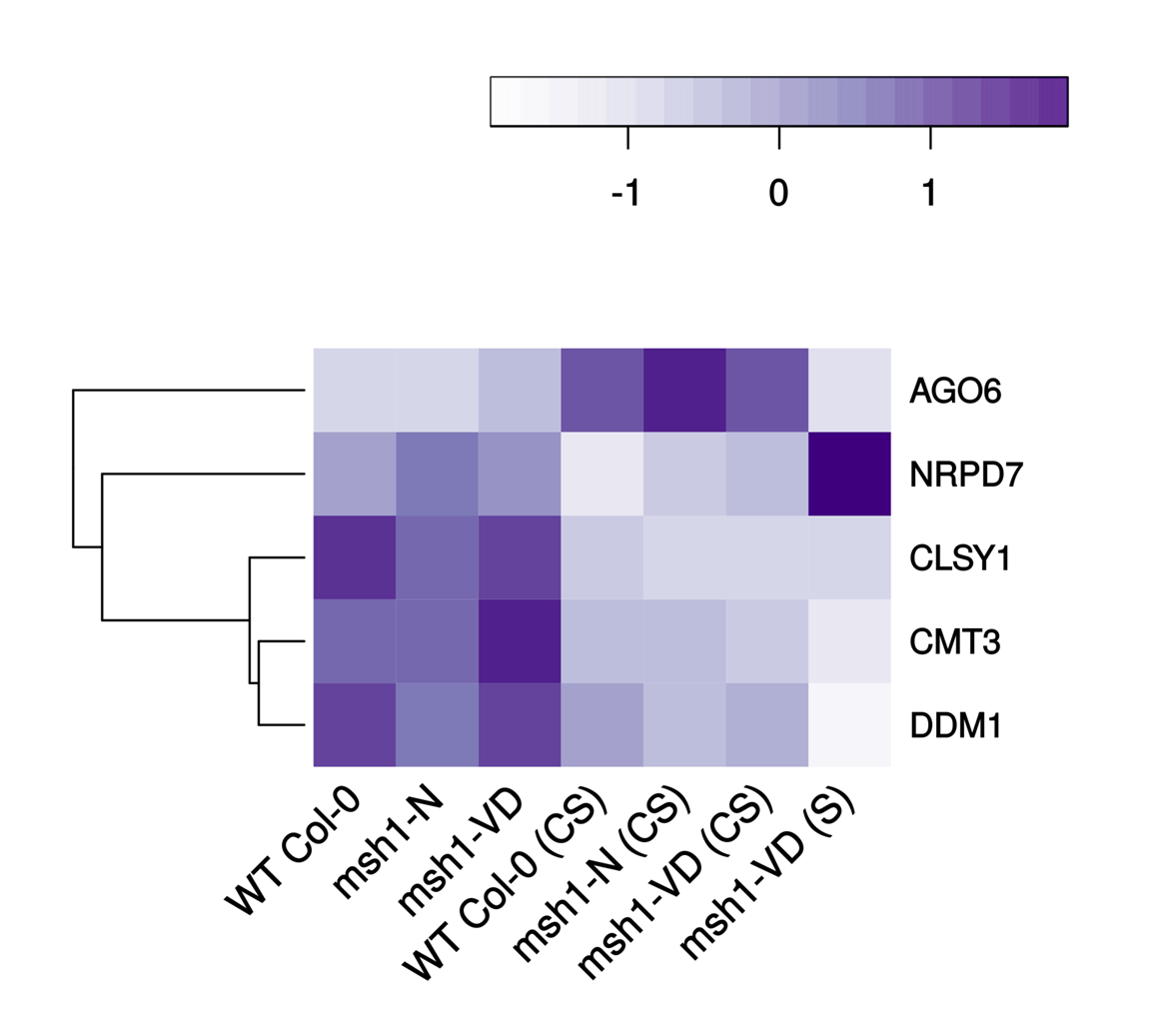

Supplement: Supplementary file 6 [file PLD3-2-e00079-s006.png]

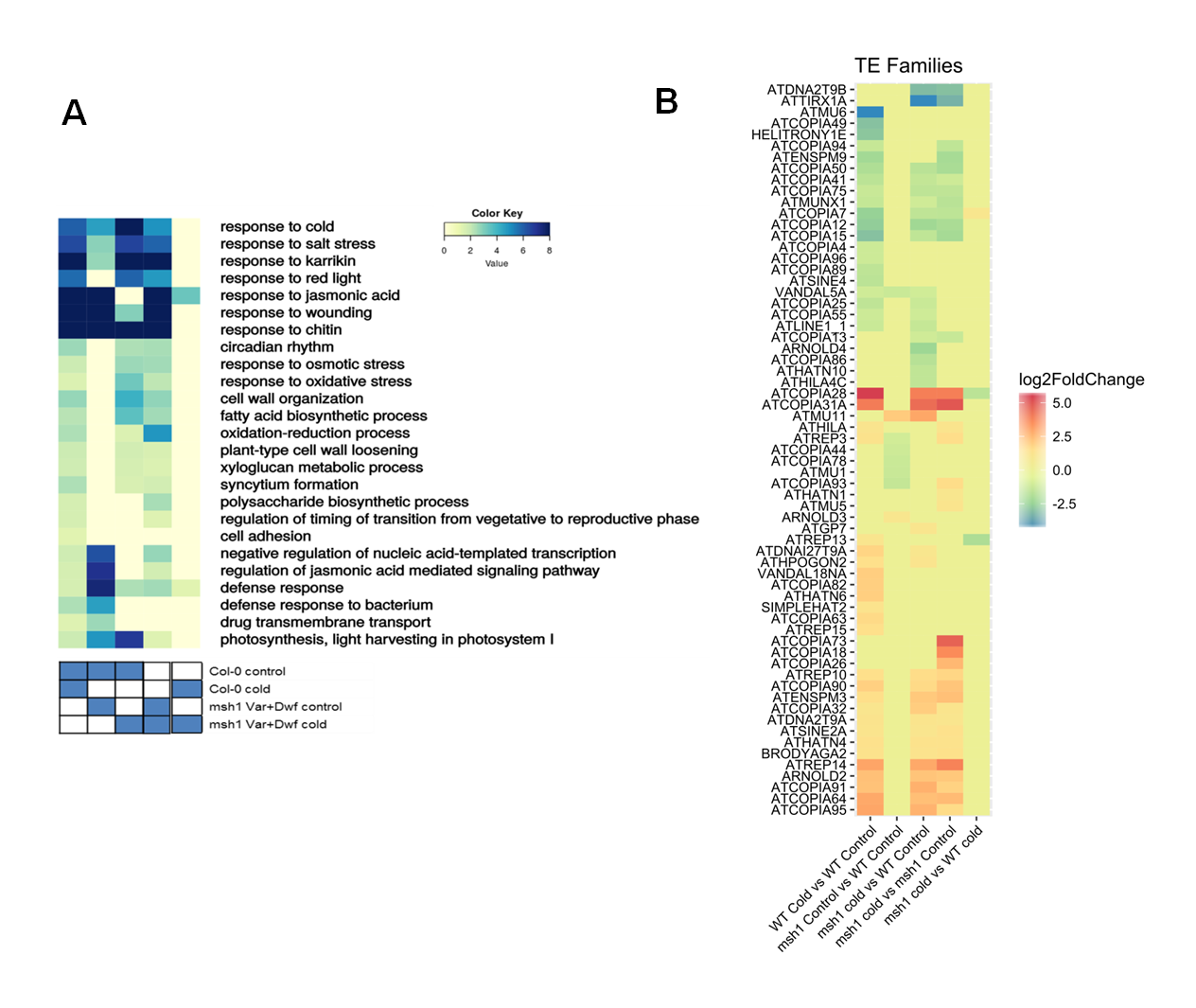

Supplement: Supplementary file 7 [file PLD3-2-e00079-s007.png]

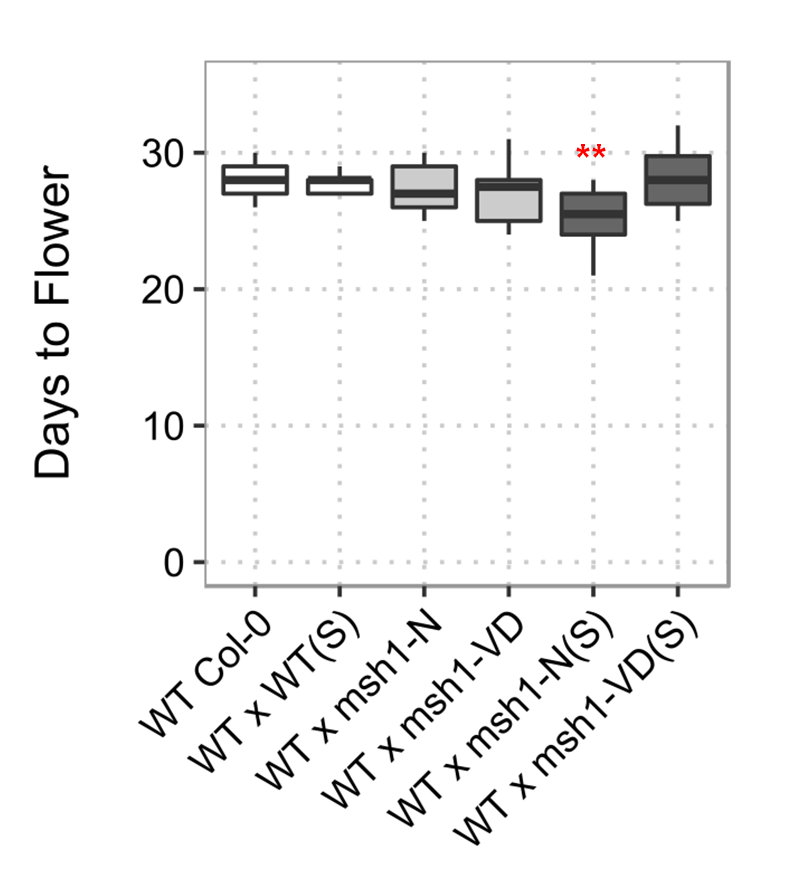

Supplement: Supplementary file 8 [file PLD3-2-e00079-s008.png]
