# Supplementary material for: Epigenomic plasticity of Arabidopsis msh1 mutants under prolonged cold stress
Source: Plant Direct. 2018 Aug 29;2(8):e00079. doi: 10.1002/pld3.79 (PMC6508824; doi:10.1002/pld3.79)
Supplement: Supplementary file 14 [file PLD3-2-e00079-s014.pdf]

Reviewer #1:

The authors have addressed my previous comments and I am in favor of publication subject to a small number of minor changes (listed below).

ln. 116. "two independent msh1 mutants". Please clarify - only one msh1 mutant is described above (SAIL_877_F01). What was the source of the second, independent mutant?

We would like to thank the reviewer for catching this. We have removed the term independent in line 116

ln. 190. "Two msh1 mutants" > Two selections from the msh1 parental mutant.

The term "selections from the .." is added in line 190 to make it clear that the lines are derived from the same parental *msh1* mutant.

ln. 283. "We also detected 2592 CG-DMRs, 109 CHG-DMRs and 2658 CHH-DMRs induced by cold stress alone in the wild type", followed by "This implies that cold stress predominantly affects CHH methylation, more than CG and CHG methylation,". I'm still confused by this. The number of CG v. CHH DMRs induceed by cold stress is approximately equal, so how does it imply cold affects CHH more? Please clarify in the manuscript.

Here we were referring to the difference in magnitude of changes in the symmetric and non-symmetric DNA methylation in msh1 mutants vs wildtype and cold stressed wild type vs control wildtype comparisons. Since our wordings were confusing, we have changed the writing in lines 283-288 to make this clear.

"We also detected 2592 CG-DMRs, 109 CHG-DMRs and 2658 CHH-DMRs induced by cold stress alone in the wild type. The magnitude of CG and CHG changes in the *msh1* mutant was four times higher than changes induced by cold stress alone in wild type, while the magnitude of CHH changes were similar. This implies that relative to CG and CHG methylation, cold stress disproportionately affects CHH methylation, consistent with previous reports of methylome behavior under low temperature (Dubin et al., 2015)."

ln. 358. Define F2:3 - it is not obvious what this means.

We have added sentences in the methods (line 94-95) and results (line 358-359) describing the F2:3 lines
